# Supplementary material for: A click chemistry amplified nanopore assay for ultrasensitive quantification of HIV-1 p24 antigen in clinical samples
Source: Nat Commun. 2022 Nov 11;13:6852. doi: 10.1038/s41467-022-34273-x (PMC9651128; doi:10.1038/s41467-022-34273-x)
Supplement: Supplementary file 1 — Supplementary Information [file 41467_2022_34273_MOESM1_ESM.pdf]

Supplementary Information

A Click Chemistry Amplified Nanopore (CAN) Assay for Ultrasensitive Quantification of HIV-1 p24 Antigen in Clinical Samples

Xiaojun Wei[1,2], Xiaoqin Wang[2], Zehui Zhang[1], Yuanyuan Luo[3], Zixin Wang[3], Wen Xiong[2], Piyush K. Jain[4,5,6], John R. Monnier[2], Hui Wang[3], Tony Y. Hu[7,8], Chuanbing Tang[3], Helmut Albrecht[9,10], and Chang Liu[1,2]*

[1]Biomedical Engineering Program, University of South Carolina, Columbia, SC 29208, USA

[2]Department of Chemical Engineering, University of South Carolina, Columbia, SC 29208, USA

[3]Department of Chemistry and Biochemistry, University of South Carolina, Columbia, SC 29208, USA

[4]Department of Chemical Engineering, University of Florida, Gainesville, FL 32611, USA

[5]Department of Molecular Genetics and Microbiology, University of Florida, Gainesville, FL 32610, USA

[6]UF Health Cancer Center, University of Florida, Gainesville, FL 32608, USA

[7]Center for Cellular and Molecular Diagnostics, Tulane University School of Medicine, New Orleans, LA 70112, USA.

[8]Department of Biochemistry and Molecular Biology, Tulane University School of Medicine, New Orleans, LA 70112, USA

[9]Department of Internal Medicine, School of Medicine, University of South Carolina, Columbia, SC 29209, USA

[10]Center of Infectious Diseases Research and Policy, Prisma Health, Columbia, SC 29203, USA

* Address correspondence to: changliu@cec.sc.edu

Supplementary Methods

Reagents and materials

Copper nitrate ($Cu(NO_3)_2$, 99%), potassium chloride (KCl, 99.99%), Tris hydrochloride (Tris-HCl), 1-Azidoadamantane (AA, 97%), 4-(2-Hydroxyethyl)piperazine-1-ethanesulfonic acid (HEPES, ≥ 99.5%), (Ethylenedinitrilo) tetraacetic acid (EDTA, ≥ 99%), ascorbic acid (≥ 99%), acetonitrile (99.8%), cucurbit[6]uril hydrate (CB[6]), bovine serum albumin (BSA, lyophilized powder, ≥ 98%), copper oxide (CuO nanopowder, particle size <50 nm), reduced L-glutathione (GSH, ≥ 98.0%,) Copper sulfate ($CuSO_4$, ≥ 99.0%), ascorbic acid, streptavidin protein, α-HL from *staphylococcus aureus* (lyophilized powder, protein ~60% by Lowry, ≥ 10,000 units/mg protein), n-pentane (anhydrous, ≥ 99%), hexadecane (Reagent Plus, 99%), 2-(N-morpholino) ethanesulfonic acid (MES, ≥ 99.5%), 1-(3-Dimethylaminopropyl)-3 ethylcarbodiimide hydrochloride (EDC, ≥ 98%), and N-hydroxysuccinimide (NHS, ≥ 97%) were purchased from Sigma-Alrich. Single stranded DNA with the sequence of 5'-CCCCCCCCCCT*CCCCCCCCCC-3' (T* indicates alkyne-modified thymine) was purchased from Sangon Biotechnology Co. Ltd. (Shanghai, China) and purified by high performance liquid chromatography (HPLC). Micro Bio-Spin P6 gel columns were purchased from Bio-Rad (Hercules, CA), and were pre-equilibrated three times with 200 μL deionized (DI) water prior to use. Lipid containing 1,2-Diphytanoyl-sn-glycero-3-phosphochline (4ME 16:0 PC) were purchased from Avanti Polar Lipids (Alabama, USA). Recombinant HIV-1 p24 protein (Expressed from *Escherichia coli*, > 90% SDS-PAGE), anti-HIV-1 p24 antibody [5] (capture antibody), anti-HIV-1 p24 antibody [38/8.7.47] (detection antibody), biotin anti-HIV-1 p24 antibody, HIV-1 p24 ELISA kit (reactivity: human), and streptavidin conjugation kit (Lightning-Link) were purchased from Abcam (USA). Dynabeads™ M-270 carboxylic acid (2×10^9 beads mL^{-1}, supplied in purified water) was obtained from Thermo Fisher Scientific (USA). All work solutions were prepared using DI water from a Milli-Q water purification system (resistivity 18.2 MΩ/cm, 25°C, Millipore Corporation) and were filtered through 0.02 μm filters before use.

Characterization

Concentrations of Cu/Fe ions were determined by inductively coupled plasma-optical emission spectroscopy (ICP-OES) analyses (Perkin Elmer Optima 2000 DV). The ^1H nuclear magnetic resonance (NMR) spectra of reactants and achieved PEG linkers were recorded at 298 K in deuterated solvents using Bruker AVANCE 400 MHz NMR spectrometer. A Thermo Q-Exactive mass spectrometer was used to characterize the DNA modification. The steady-state luminescence spectra of Cu nanoclusters were recorded and measured using a fluorescence spectrometer with a Xe lamp. Measurements of the fluorescence signal from Cu nanoclusters were obtained *via* the SpectraMax microplate reader. Transmission electron microscopy (TEM) images were acquired with a H-7800 electron microscope operating at an accelerating voltage of 120 kV. Thermo gravimetric analysis (TGA) was performed using a NETZSCH TG209F3 thermogravimetric analyzer. Dynamic light scattering (DLS) measurements were carried out at 25°C on a Nano-ZS90 (Malvern) equipped with a diode (solid state) laser (λ = 633nm). Fourier transform infrared (FTIR) spectra were obtained at room temperature using a Nicolet iS10 (Thermo Scientific) spectrometer.

Buffer preparation

Assay buffer consists of 0.1 M NaCl, 0.025% Tween 20, 0.1% BSA, and 10 mM Na_2HPO_4 (pH 7.2). Washing buffer consists of 0.15 M NaCl and 10 mM Na_2HPO_4. Glycine buffer was obtained by dissolving glycine (0.375 g) in 40 mL of H_2O and adjusting the pH to 2.0 with concentrated HCl, then added the 0.05 mL Triton X-100 and adjusted the volume to 50 mL with H_2O. Carbonate buffer was obtained by dissolving anhydrous sodium carbonate (0.088 g) and sodium bicarbonate (0.7728 g) in 50 mL H_2O, then brought to 200 mL with H_2O with final pH of 9.0.

Nanopore work solution

KCl (22.37 g) and Tris-HCl (0.121 g) were first dissolved in 80 mL DI water. NaOH (2 M) and HCl (2 M) were used to adjust the pH of the solution to 8.0. The solution was then diluted with DI water to 100 mL to afford the final work solution with 3M KCl and 10mM Tris-HCl at pH 8.0.

Magnetic beads functionalization with capture antibodies

Magnetic beads (MBs, 100 µL) were first washed three times using 200 µL MES (25 mM, pH 5.0), and then dispersed in MES (25 mM, pH 5.0) with 50 µL EDC (50 mg/mL) and 50 µL NHS (50 mg/mL). After a 30-min incubation at room temperature with vortex, MBs were separated from the supernatant and washed three times with MES solution. Carboxyl activated MBs was then mixed with capture antibodies (100 µg, 1mg/mL, undiluted anti-HIV-1 p24 antibody [5], Abcam, Cat#: ab63958) in 100 µL MES and incubated for 30 minutes at room temperature with rotation. After this, antibody functionalized MBs were separated and further incubated with 100 µL Tris-HCl (50 mM, pH 7.4) for 20 minutes to quench excessive activated carboxylic acid groups. Finally, antibody functionalized MBs were washed four times with 100 µL phosphate buffer saline (PBS, pH 7.4), blocked by vortexing in 50 µL 0.05% BSA for 10 minutes, and resuspended in 120 µL PBS for further use.

Preparation of glutathione (GSH) stabilized blue fluorescent Cu nanoclusters (NCs)

The GSH-stabilized blue fluorescent CuNCs were prepared by a thermo-reduced method.[1] In a typical experiment, GSH (5 mL, 50 mg/mL) was mixed with $CuSO_4$ solution (5.0 mL, 10 mM) till the solution became cloudy. Next, NaOH solution (100 µL, 1 M) was added dropwise with vigorous stirring until the mixture solution changed to clear. Then the mixture was kept stirring at 80°C for 12 h until the color changed from pale blue to purple. The final solution of CuNCs was then gradually cooled down to room temperature. The resulting concentrated CuNCs were precipitated by addition of isopropanol (volume ratio of isopropanol and sample solution is 3:1), collected through centrifugation at 3500 x g for 5 min, washed with isopropanol for three cycles, and finally redispersed in 10 mL PBS for further experiments.

Preparation of glutathione (GSH) stabilized red fluorescent Cu nanoclusters (NCs)

The GSH-stabilized red fluorescent CuNCs were prepared through a slightly modified method from literature.[2, 3] In a typical preparation, 5.0 mL mixture solution of GSH (50 mg/mL) and ascorbic acid (10 mg/mL) was added into 5.0 mL of $CuSO_4$ solution (10 mM) under vigorous stirring. The change from a transparent solution to a white suspension liquid implied that a hydrogel was formed due to the coordination between Cu ions and various functional groups of thiol moieties as well as carboxylate

groups in GSH. Then, 0.4 mL of NaOH solution (1 M) was added dropwise until the turbid liquid turned to transparent light yellow. The corresponding pH value was controlled to 4.5-5.0. Subsequently, the mixture solution was stirred vigorously at 45 °C for 1 h and cooled to room temperature to obtain CuNCs with red emission photoluminescence. The resultant CuNCs were precipitated by isopropanol (volume ratio of isopropanol and sample solution is 3:1), collected by centrifugation at 3500 x g for 5 min, washed with isopropanol for three cycles, and finally redispersed in 10 mL PBS for further experiments.

Bioconjugation of streptavidin to GSH-stabilized CuNCs

The conjugation of GSH stabilized CuNCs with streptavidin requires activation of the carboxyl groups of GSH which was performed using EDC/sulfo-NHS protocol.[4] Typically, CuNCs were first mixed with EDC (10 mM) and sulpho-NHS (20 mM) in PBS buffer for 30 min to activate the carboxyls of GSH on CuNCs. Then, activated CuNCs were washed with glycine buffer, and added to 200 uL of streptavidin protein (1 mg/mL) prepared in carbonate buffer. After an incubation period of 24 hours at 25°C followed by multiple washes with glycine buffer, streptavidin conjugated CuNCs were obtained and diluted to 1 mM/mL in PBS. The final products were kept at 4°C for storage for further experiments.

Quantification of p24 antigens in human serum using fluorescent CuNC assay

Workflow of the CuNCs based assay for quantification of HIV-1 p24 antigen in human serum is shown in Supplementary Fig. 1. Antibody-coated MBs (20 μL in PBS) were washed with 500 μL assay buffer for three times and dispersed in 500 μL diluted healthy donor serum (human serum: assay buffer = 1:1, v/v). Then the solution was introduced into a black 96-well black plate with transparent bottom. To establish a calibration curve, standard samples were made by adding various amount of p24 antigens to sera to reach final concentrations of 1, 5, 10, 20, 50, 100, 500, 1000 pg/mL, and vortexed for 30 minutes at 25°C. Next, biotin anti-HIV-1 p24 antibody (100 μL, 4mg/mL, Undiluted, Abcam, Cat#: ab68617) were mixed with each standard sample and vortexed for 30 minutes at 25°C. After forming sandwich structures, MBs were magnetically separated and washed three times with washing buffer. Streptavidin conjugated CuNCs (100 μL per well) were added to the above antibody-antigen-antibody complex and the mixture was incubated and vortexed for 30 minutes at 25°C. A final round of washing was performed with PBS buffer for 5 times to avoid nonspecific interactions and to reduce background noise. Finally, measurements of the fluorescence signal from CuNCs were obtained using a SpectraMax microplate reader (excitation at 370 nm and emission at 440 nm for blue fluorescent CuNCs based assay; excitation at 360 nm and emission at 630 nm for red fluorescent CuNCs based assay).

Quantification of p24 antigens in human serum using ELISA kit

To establish a calibration curve, each diluted serum sample (500 μL, human serum: assay buffer = 1:1, v/v) was mixed with different amount of p24 antigens to reach final concentrations of 0, 0.5, 1, 5, 10, 50, 100, 300, 1000 pg/mL, respectively. p24 in human serum was quantified using a commercial kit according to the manufacturer's specifications. Briefly, 50 μL of sample and 50 μL of the Antibody Cocktail were added to each well, then the plate was sealed and incubated for 1 hour at room temperature on a plate shaker set to 9 x g. After washing three times with 350 μL 1×Wash Buffer, 100

L of TMB Solution was added to each well and incubated for 10 minutes in the dark on a plate shaker (14 x g). The reaction was then terminated by adding 100 µL Stop Solution to each well before recording its optical density (OD) at 450 nm using a Spectra Max 190 Microplate Reader (Absorbance) at room temperature.

Calculation of the bilayer membrane thickness

The membrane capacitance, C_m, defines how much charge, q, is stored on two capacitor plates at a fixed membrane voltage, V_m,

$$q = C_m \cdot V_m$$

For a parallel plate capacitor, C_m is given by

$$C_m = \epsilon_0 \epsilon \frac{A}{D}$$

with vacuum permittivity $\varepsilon_0 = 8.854 \times 10^{-12}$ F/m and dielectric constant $\varepsilon \approx$ 2-4. Here, A is the area of the membrane, and D is its thickness.

Supplementary Figures

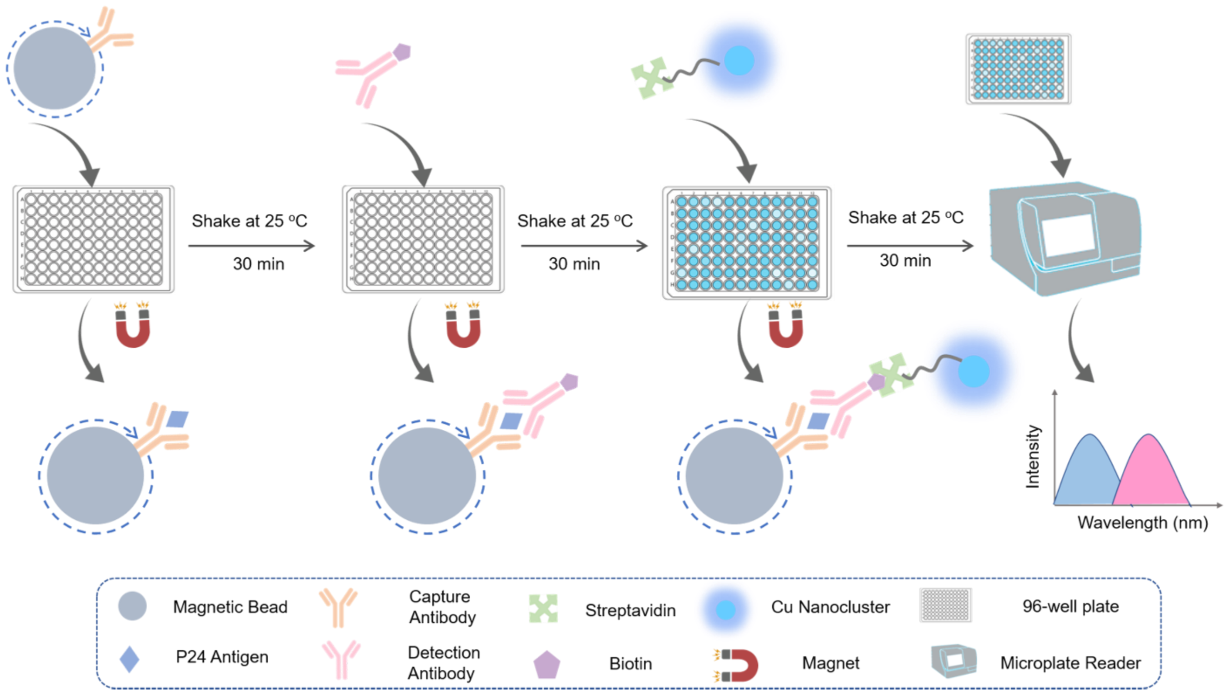

Supplementary Fig. 1 Workflow of the CuNCs based assay for quantification of HIV-1 p24 antigen in human serum: (1) Serum samples were incubated with capture antibody-modified magnetic beads and biotin-modified detection antibody to form a sandwich structure; (2) Fluorescent probes were formed by adding streptavidin conjugated CuNCs to the magnetically separated sandwich complexes; (3) Fluorescence probes were collected and measured by microplate reader for p24 antigen quantification. The illustration is not drawn to scale.

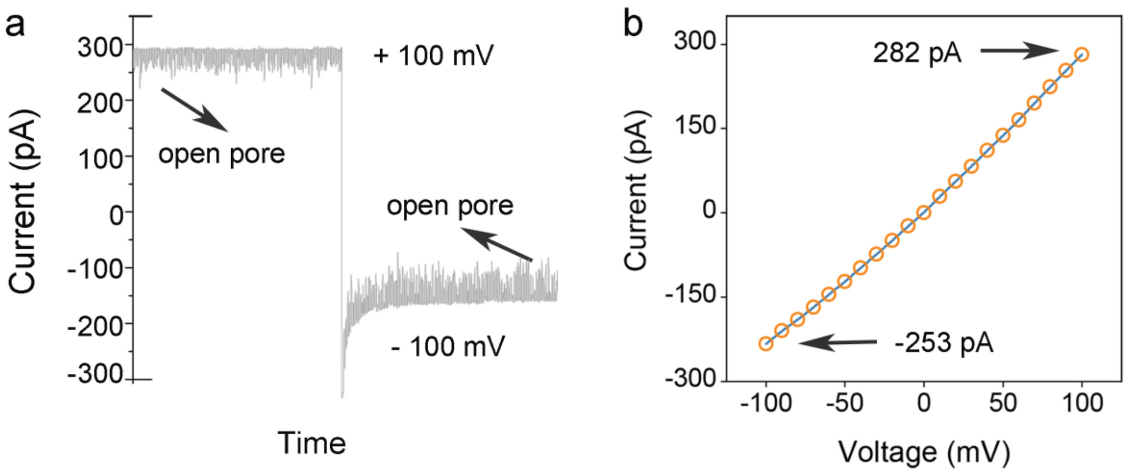

Supplementary Fig. 2 a: Corresponding raw open pore current traces at ±100 mV without any analytes in both sides of the nanopore. **b:** The I-V curve of a single open α-HL nanopore in the work solution of 3 M KCl, 10 mM Tris, pH 8.0. The slope of the fitted curve was 2.58 ± 0.02 ns. Source data are provided as a Source Data file.

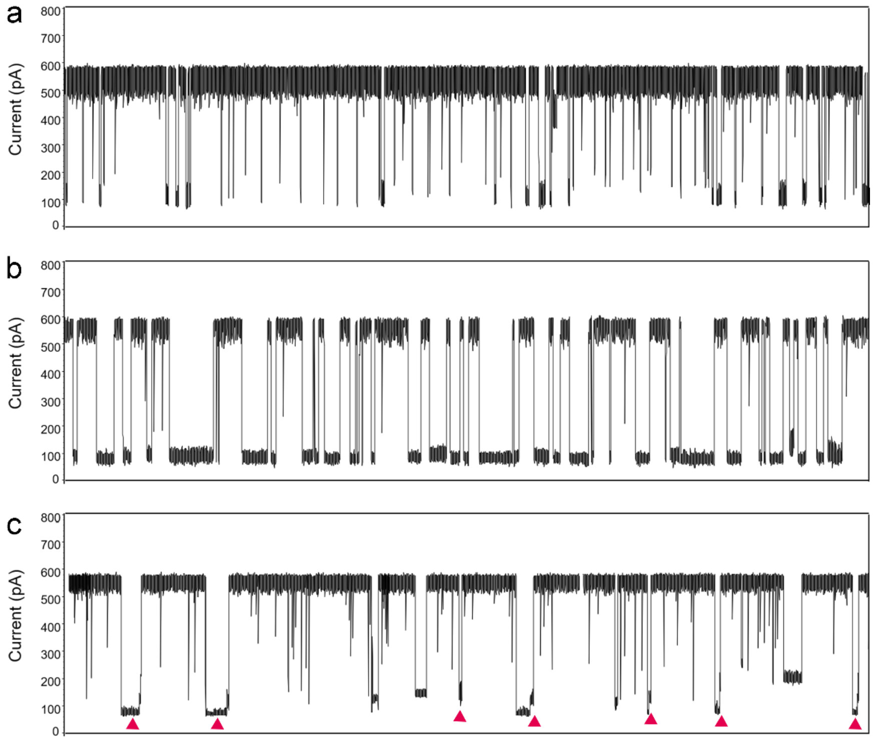

Supplementary Fig. 3 Raw current traces of (a) alkyne modified DNA, (b) DNA-AA, and (c) DNA-AA@CB[6] translocations in α-HL, respectively. Data was acquired using 3 M KCl, 10 mM Tris buffer, pH 8.0. Red triangles indicate multi-level signals.

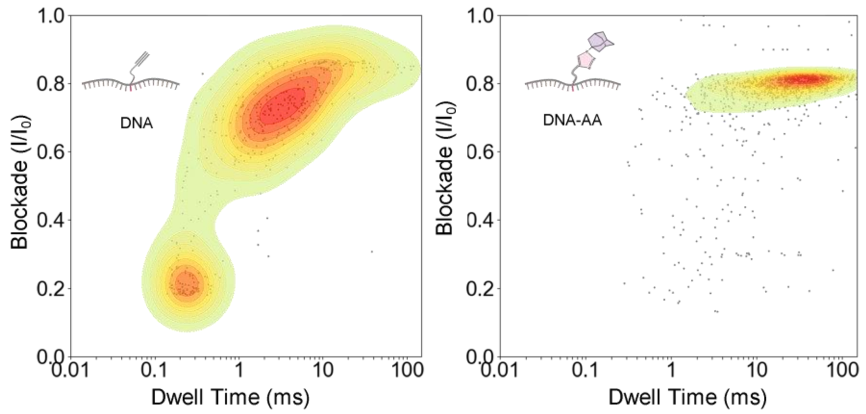

Supplementary Fig. 4 Molecular structures and corresponding contour plots depicting the blockade (I/I_0) *vs* dwell time for DNA and DNA-AA. Source data are provided as a Source Data file.

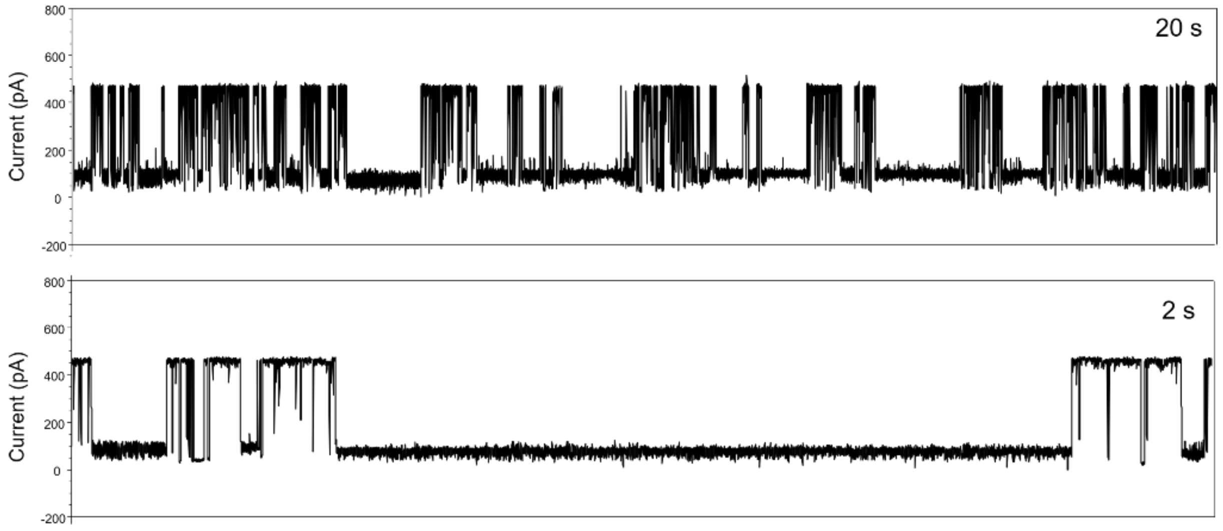

Supplementary Fig. 5 Representative time scaled (20 s and 2 s) raw current traces of CB[6] translocations. Data was acquired using 3 M KCl, 10 mM Tris buffer, pH 8.0. The final concentration of CB[6] in the *cis* side is 0.1 mM.

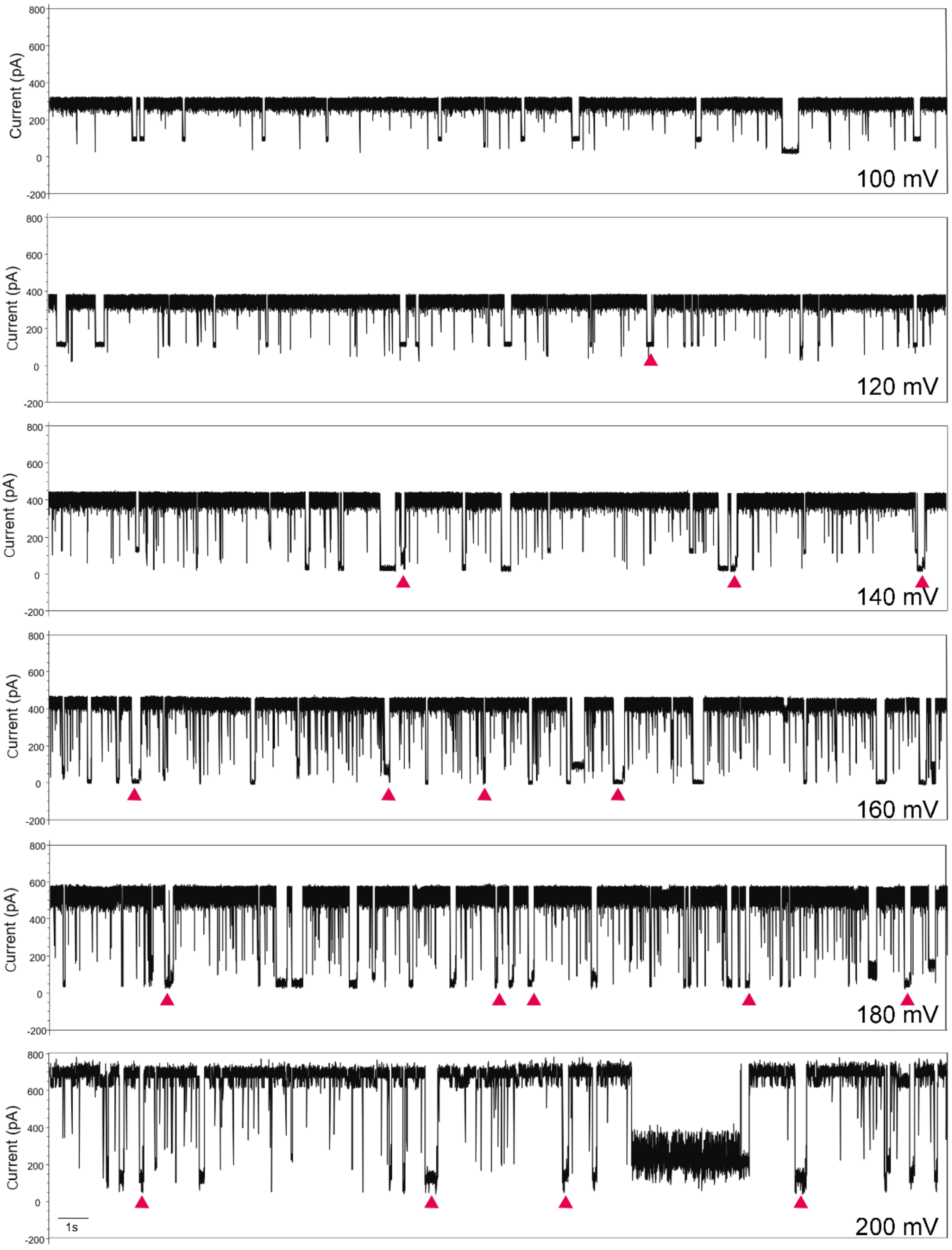

Supplementary Fig. 6 Raw current traces of probe DNA-AA@CB[6] translocations under an increasing positive transmembrane potential from 100 mV to 200 mV. Red triangles indicate multi-level signals.

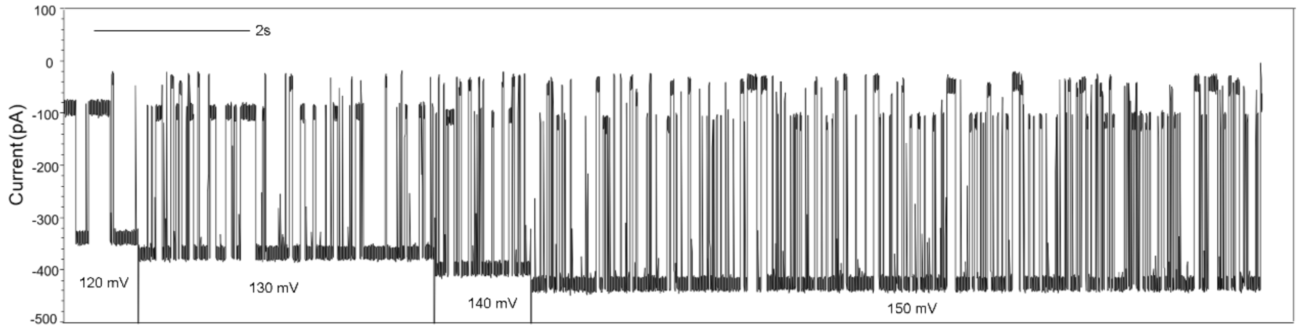

Supplementary Fig. 7 Continuous raw current traces of probe DNA-AA@CB[6] translocations under an increasing negative transmembrane potential from -120 mV to -150 mV.

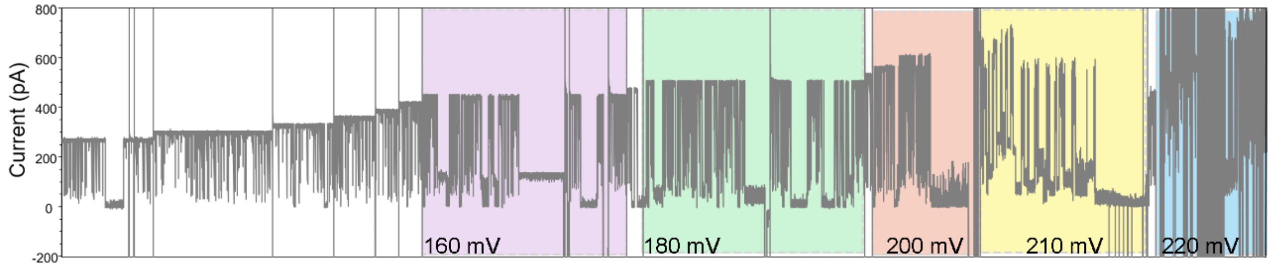

Supplementary Fig. 8 Continuous raw current traces of probe DNA-AA@CB[6] translocations under an increasing positive transmembrane potential from 100 to 220 mV (indicated by different shadow colors). Current leakage can be observed with applied potential above 200 mV.

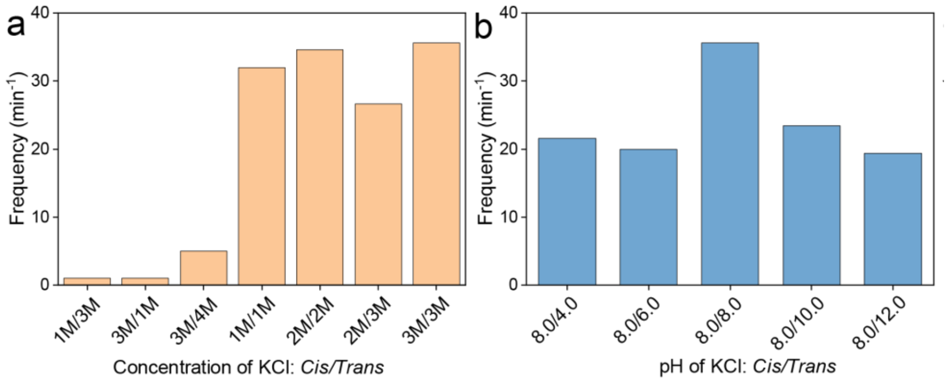

Supplementary Fig. 9 Translocation frequency of multi-level signals of probe DNA-AA@CB[6] with different (a) KCl concentration and (b) pH of the work solution. Source data are provided as a Source Data file.

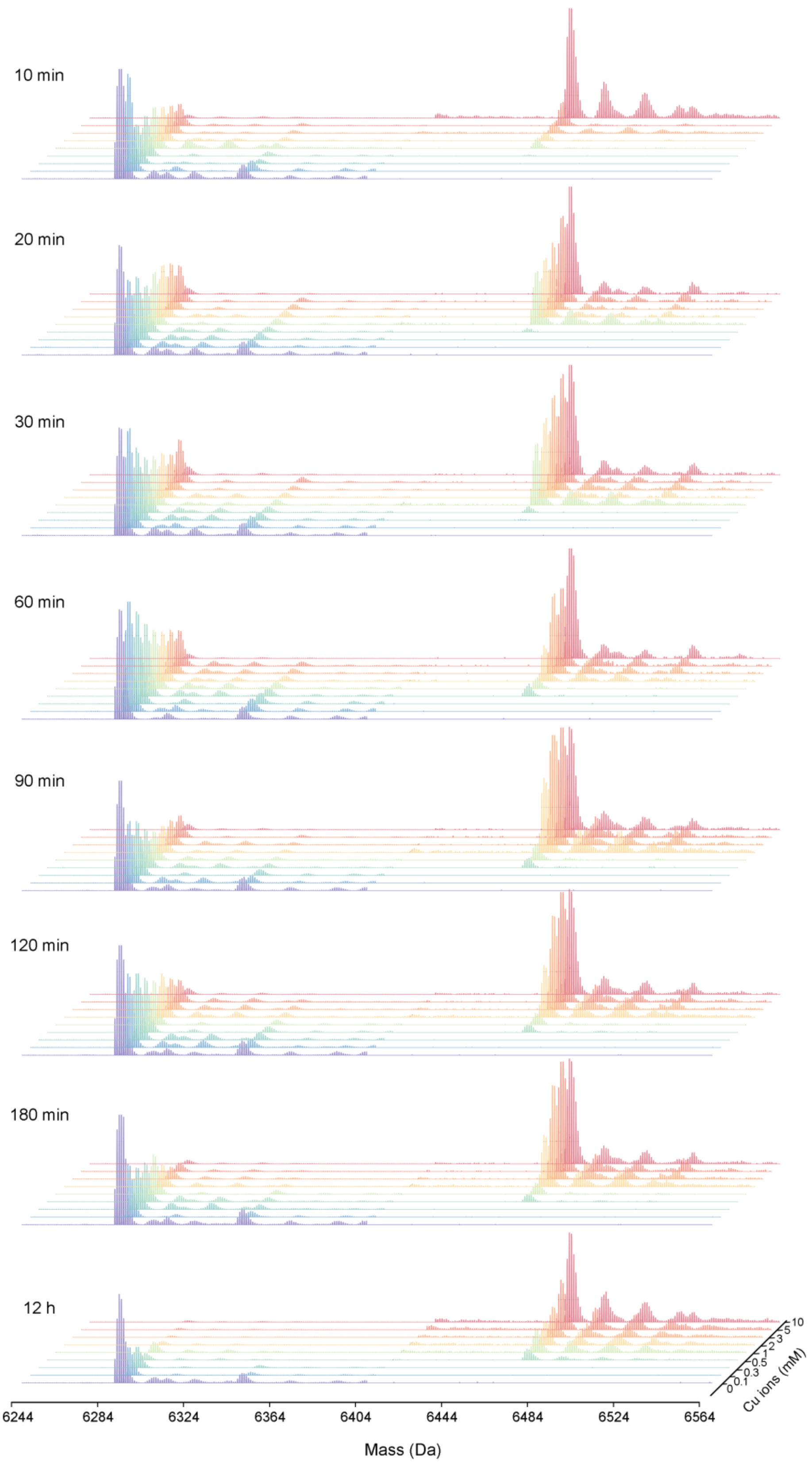

Supplementary Fig. 10 Mass spectrometry characterizations of DNA-AA obtained with different Cu^{+} ion concentrations and various reaction times. Source data are provided as a Source Data file.

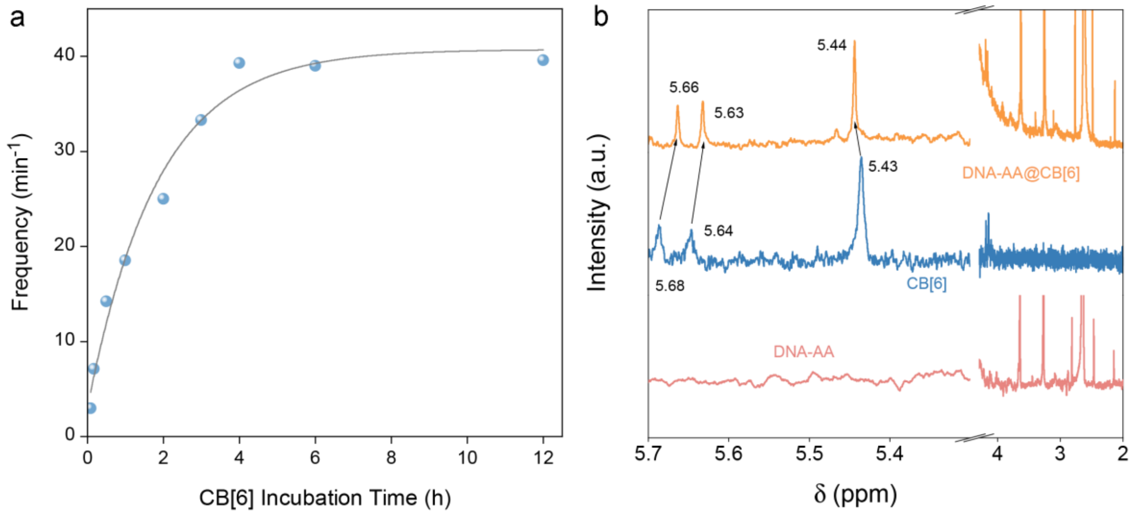

Supplementary Fig. 11 a: Multi-level signal frequency of probe DNA-AA@CB[6] as a function of the incubation time with CB[6]. **b:** ^{1}H NMR (400 MHz) spectra of DNA-AA, CB[6] and DNA-AA@CB[6]. The binding interaction between DNA-AA and CB[6] complex can be deduced via the chemical shift of protons of the CB[6] molecule from methylene (CH_2) groups (5.68 ppm and 5.43 ppm) and tertiary C–H groups (5.64 ppm). [5, 6] Source data are provided as a Source Data file.

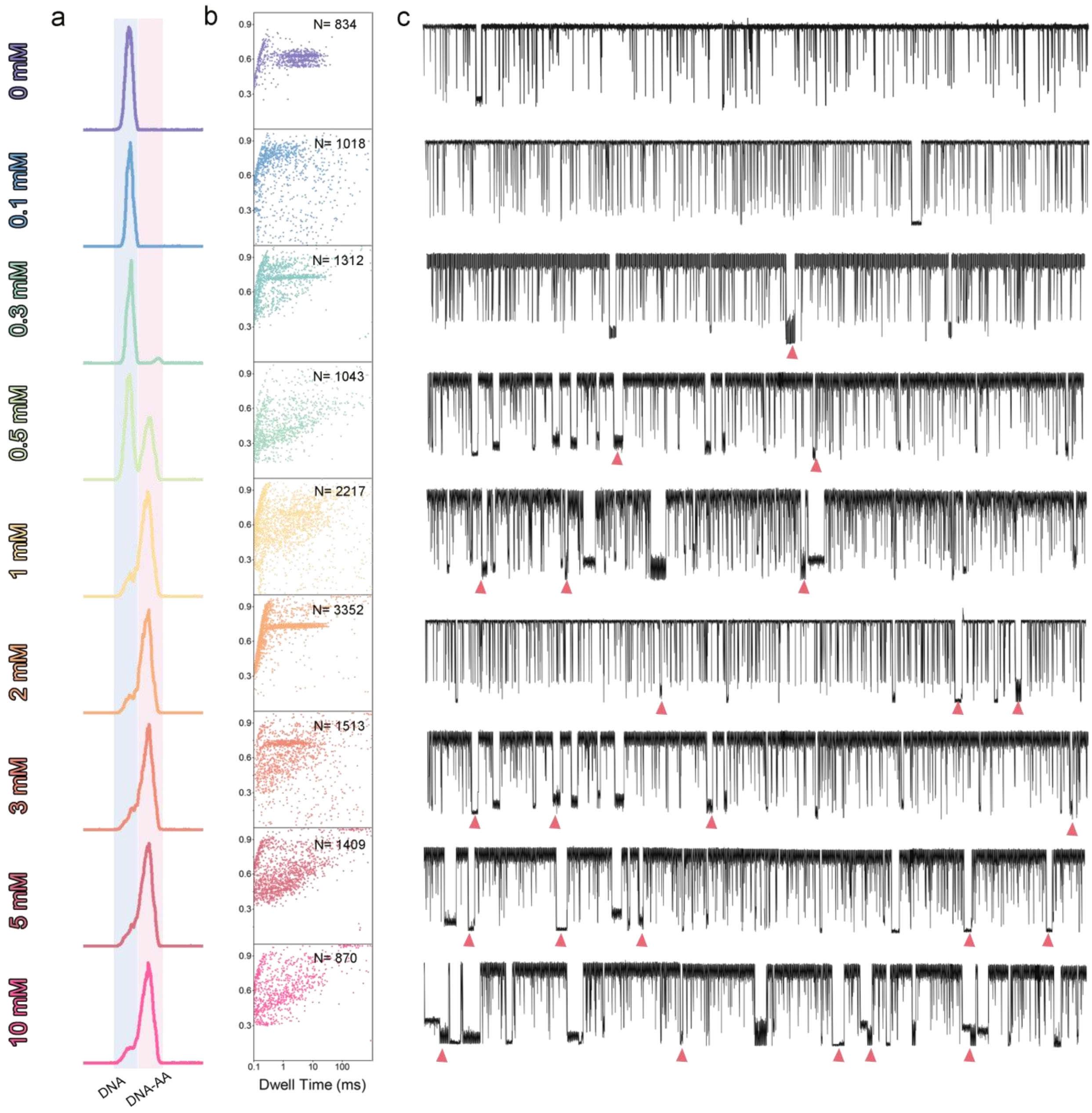

Supplementary Fig. 12 a: Distribution of subpopulations of alkyne modified DNA and DNA-AA obtained with various Cu^{+} ion concentrations characterized by HPLC retention time **b:** Corresponding two-dimensional scatter plots of translocation signals of all subpopulations (*i.e.* alkyne modified DNA, DNA-AA, and DNA-AA@CB[6]); **c:** Raw current traces of probe DNA-AA@CB[6] obtained with various Cu^{+} ion concentrations. Source data are provided as a Source Data file.

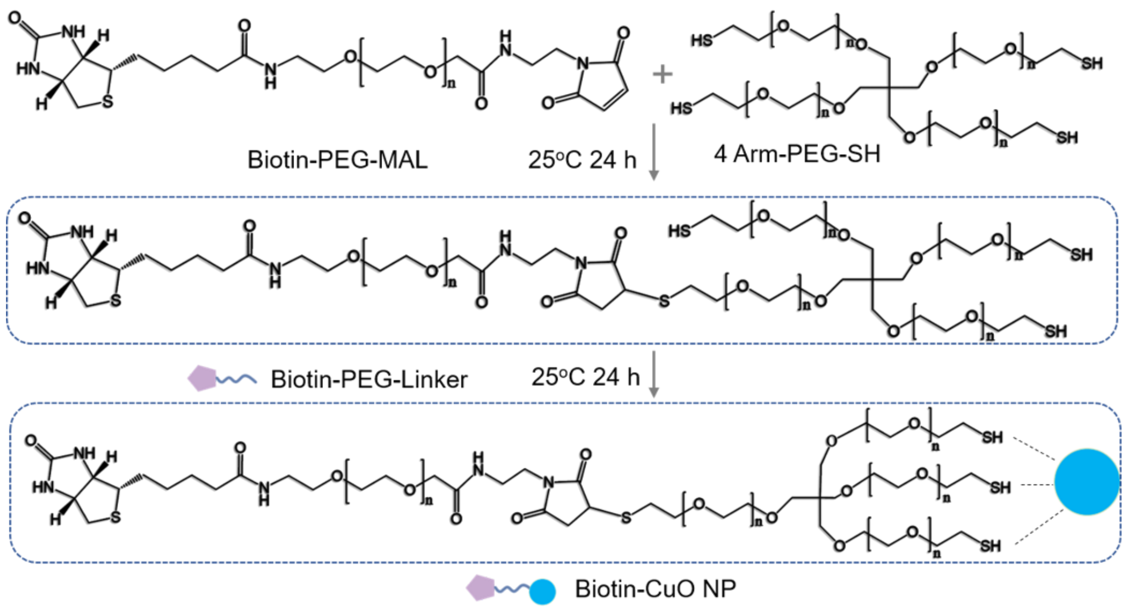

Supplementary Fig. 13 One step synthesis of biotin-PEG linkers and conjugation of biotin-PEG linkers to CuO nanoparticle surface.

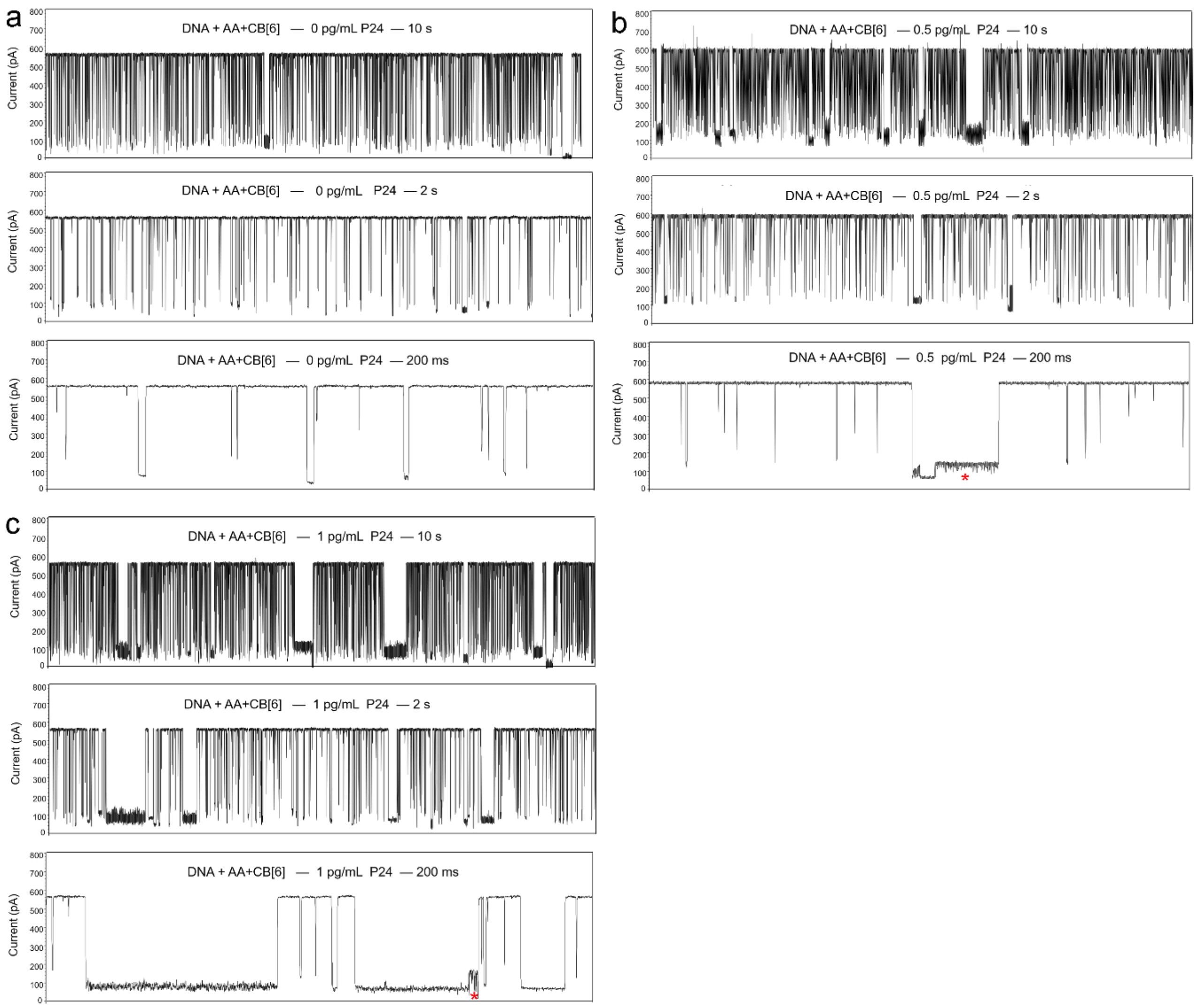

Supplementary Fig. 14 Representative time scaled (10 s, 2 s, and 200 ms) raw current traces of probe DNA translocations obtained for human serum samples spiked with (a) 0 pg/mL (b) 0.5 pg/mL and (c) 1 pg/mL p24 antigens. Data was acquired using 3 M KCl, 10 mM Tris buffer, pH 8.0. Red stars indicate multi-level signature events.

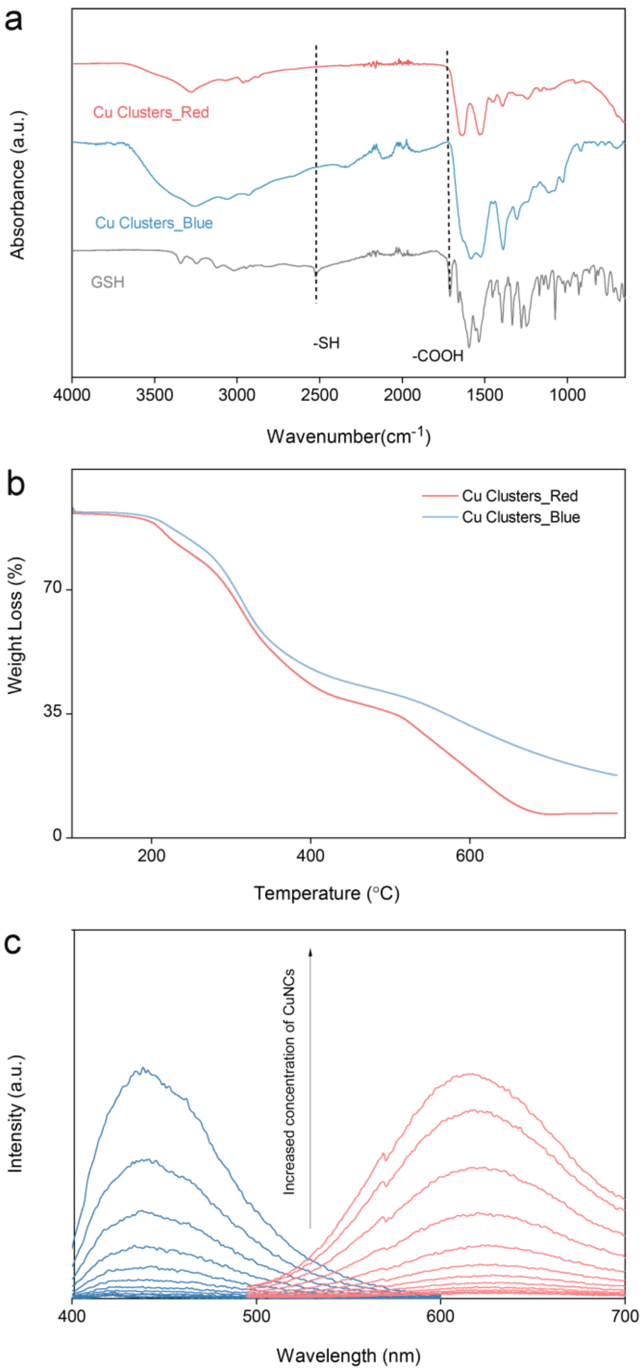

Supplementary Fig. 15 a: FTIR spectra of Cu clusters showing a typical absorption band of the carboxyl group at 1720 cm^{-1} corresponding to the weak interaction of glycine residues of GSH with Cu clusters.[7] Compared to pure GSH, the characteristic peak of SH at 2521 cm^{-1} disappeared in GSH-stabilized Cu clusters, suggesting the interaction between thiol group and Cu clusters.[1, 8] **b:** Thermogravimetric curves of Cu clusters. **c:** Fluorescence spectra of blue and red fluorescent CuNCs in aqueous solution with increasing concentrations from 0.001 to 1 mM/mL. Source data are provided as a Source Data file.

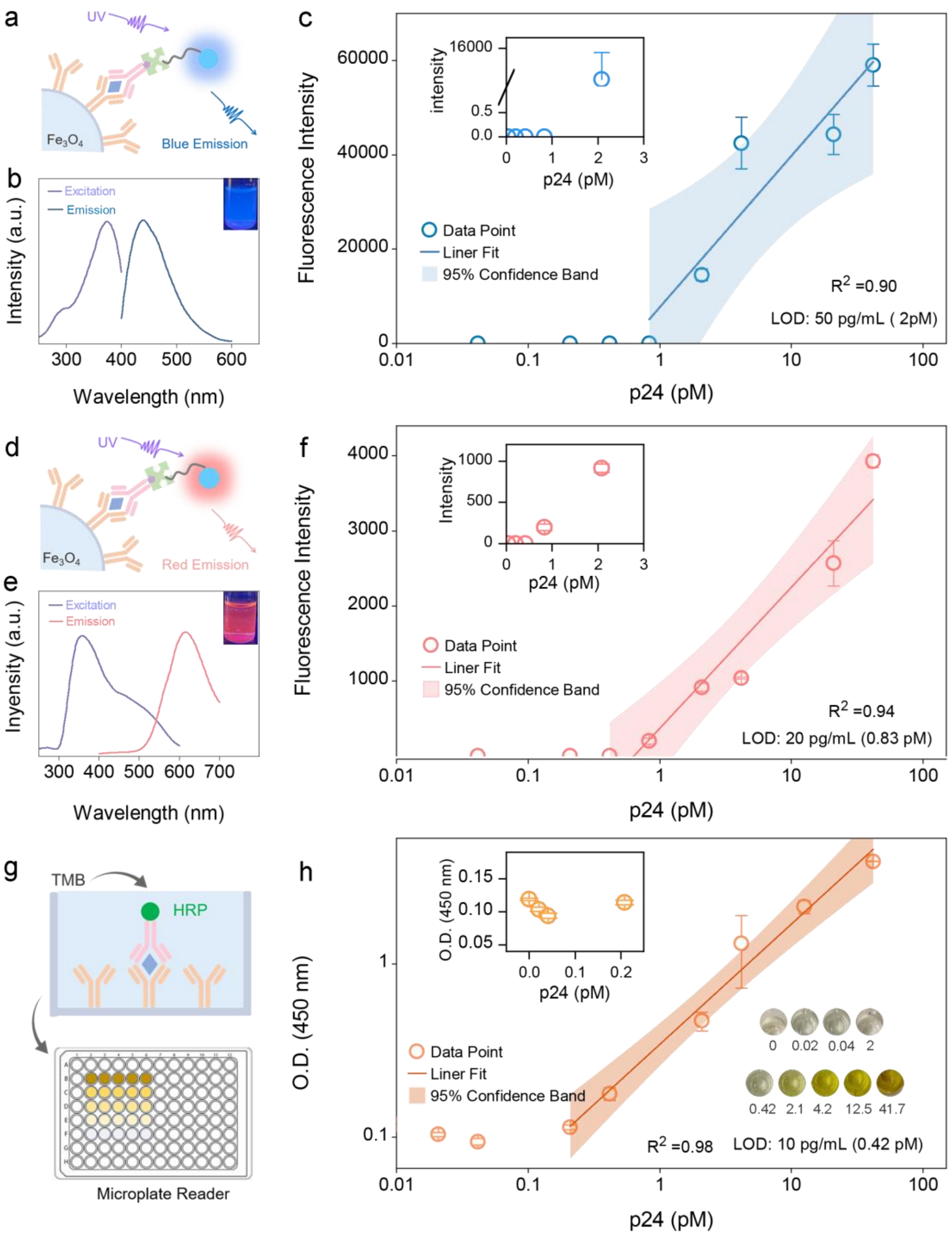

Supplementary Fig. 16 Analytical performance comparison with other p24 detection methods. **a:** Structure of the blue emission fluorescent CuNCs linked immunosorbent assay. **b:** The excitation and emission spectra of blue fluorescent CuNCs in aqueous solution recorded upon excitation at 370 nm with a Xe lamp. Inset shows the photograph of a corresponding sample under UV light (365 nm). **c:** Calibration curve of the blue emission fluorescent CuNCs-based p24 assay in human serum within the range of 0.04-46.7 pM (1-1000 pg/mL). Inset shows assay results in the range of 0.04-2.1 pM (1-50 pg/mL). **d:** Structure of the red emission fluorescent CuNCs linked immunosorbent assay. **e:** The excitation and emission spectra of red fluorescent CuNCs in aqueous solution recorded upon excitation at 360 nm with a Xe lamp. Inset shows the photograph of a corresponding sample under a UV light (365 nm). **f:** Calibration curve of the red emission fluorescent CuNCs-based p24 assay in human serum

within the range of 0.04-46.7 pM (0-1000 pg/mL). Inset shows assay results in the range of 0.04-2.1 pM (1-50 pg/mL). **g-h:** Schematic and calibration curve of an ELISA based fourth generation HIV-1 testing kit for p24 in human serum within the range of 0-46.7 pM (0-1000 pg/mL, final concentration: 0, 0.5, 1, 5, 10, 50, 100, 300, 1000 pg/mL). Insets show the correlation within lower range 0-0.2 pM (0-5 pg/mL) and corresponding pictures of ELISA results. Each data point in c, f, and h represents mean ± SD of three replicates. Solid line indicates linear regression. Shadow indicates limits of 95% confidence interval of fitted line. Source data are provided as a Source Data file.

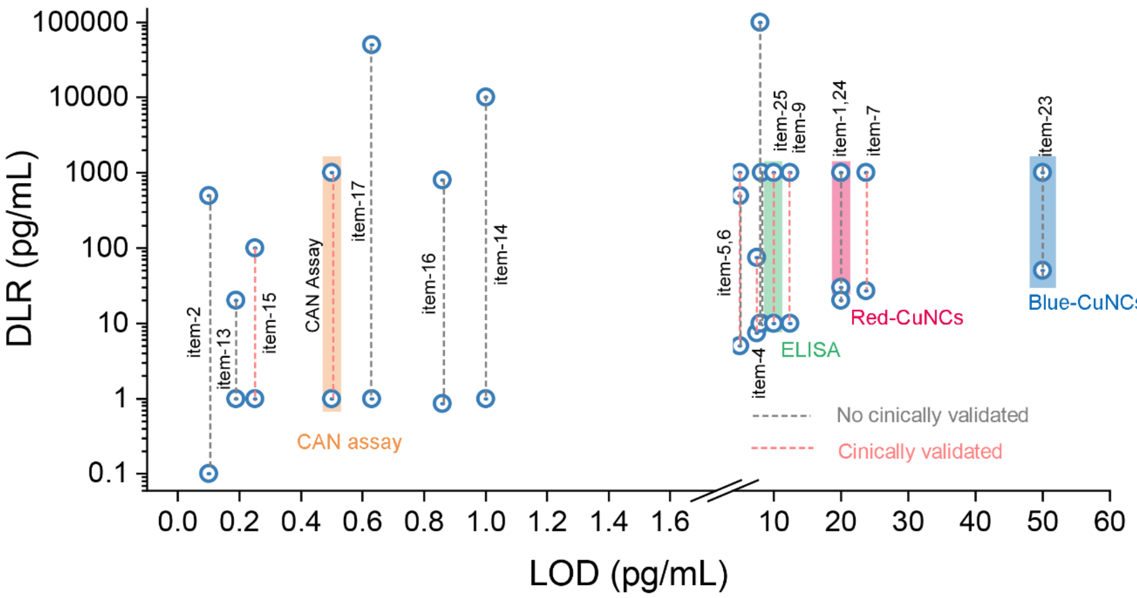

Supplementary Fig. 17 LOD and dynamic linear range (DLR) comparison of CAN assay to previously reported comparable immunoassays summarized in Table S2. Source data are provided as a Source Data file.

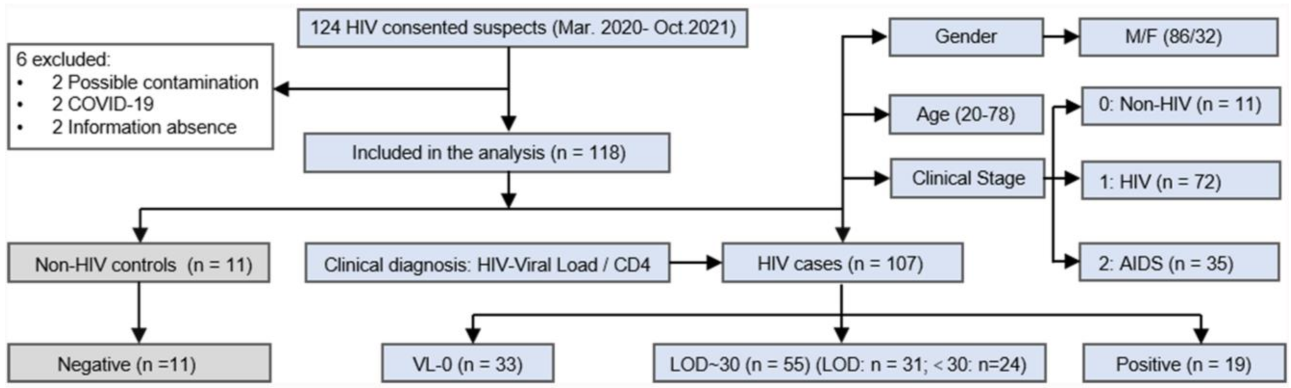

Supplementary Fig. 18 Clinical validation participant disposition flow diagram**.**

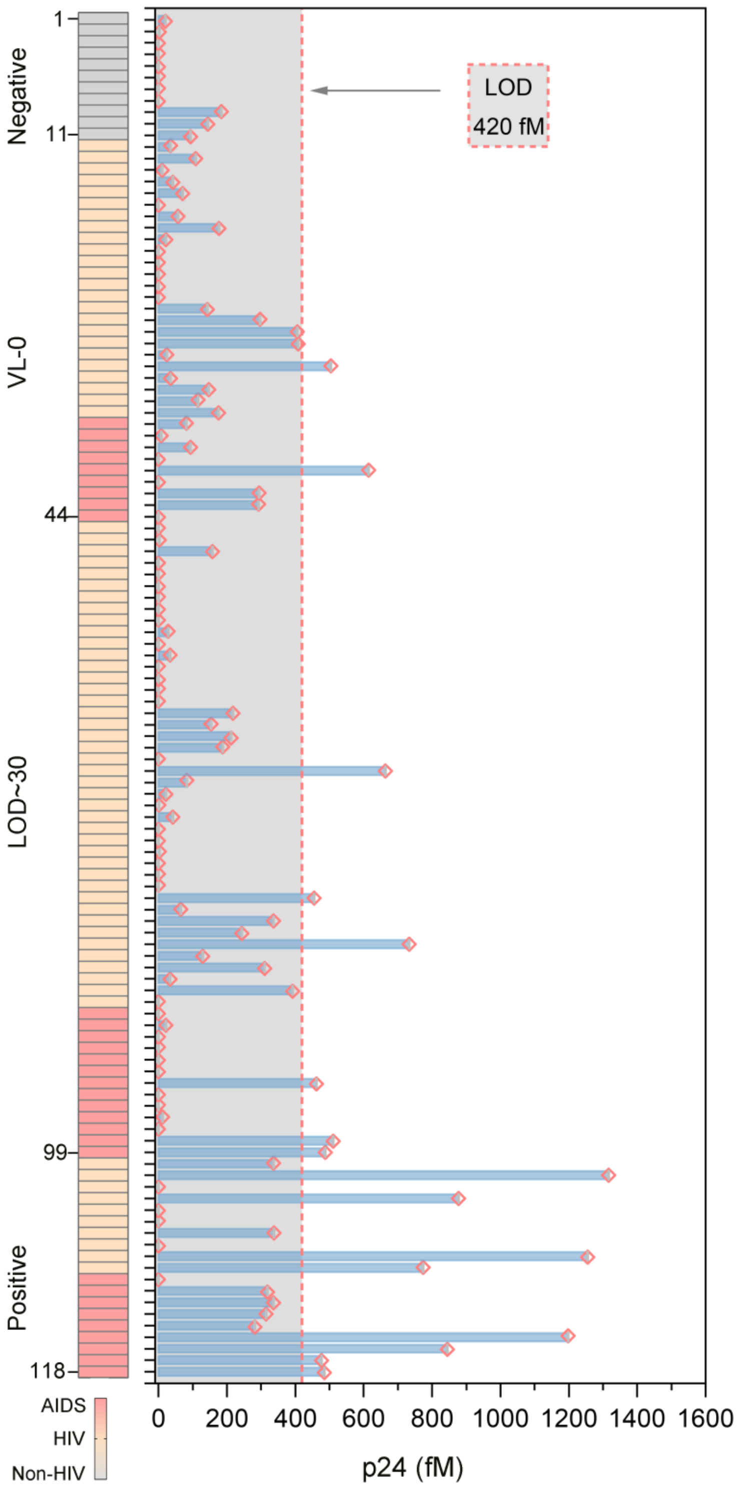

Supplementary Fig. 19 Quantitative measurement of p24 in clinical samples using ELISA, with the clinical diagnosis of each patient. The dashed line indicates the LOD of ELISA. Source data are provided as a Source Data file.

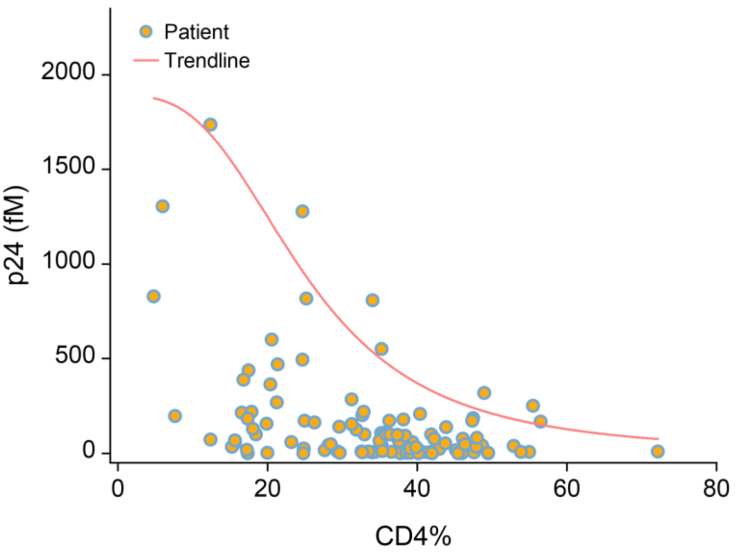

Supplementary Fig. 20 Correlation between CD4 percentage and CAN assay measured p24 concentrations. Source data are provided as a Source Data file.

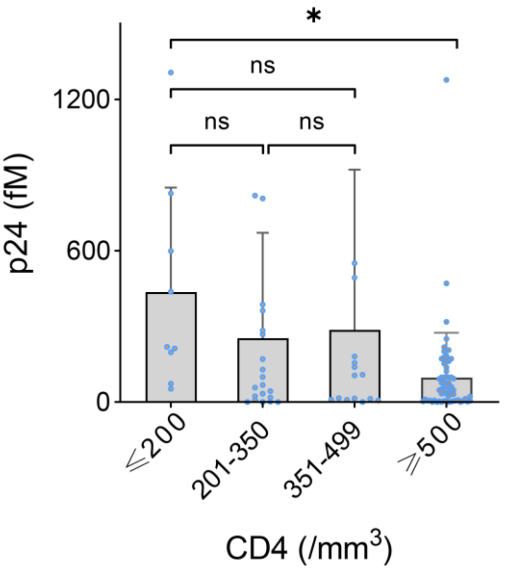

Supplementary Fig. 21 Densitometric analysis of p24 concentrations across different groups classified by CD4 counts: ≤200/mm^3 (n = 9 individuals), 201-350/mm^3 (n = 21 individuals), 351-499/mm^3 (n = 15 individuals), and ≥500/mm^3 (n = 62 individuals). Bar graph data represents mean ± SD of p24 value of all individuals in each group. p values were calculated by one-way ANOVA with post-hoc Tukey tests. * indicates p <0.05, ns indicates no significant difference. p (≤200 *vs.* 201-350) = 0.55, F (9,21) = 253.3; p (≤200 *vs.* 351-499) = 0.73, F (9,15) = 286.8; p (201-350 *vs.* 351-499) = 0.99, F (21,15) = 286.8; p (≤200 *vs.* ≥500) = 0.03, F (9,62) = 97.64; Source data are provided as a Source Data file.

Supplementary Tables

Table 1. Capture rate of multi-level signals obtained from parallel experiments for various p24 concentrations in spiked human sera.

Route	p24 pg/mL	p24 pM	Capture Rate (min^{-1})					Recording Time (s)	Counted Events
			Pore 1	Pore 2	Pore 3	Pore 4	Pore 5		
Route II	0	0	0	0.83	0.6	0.6	0	281	2
	0.5	0.021	2.65	2.35	2.26	2.2	2.1	579.2	22
	1	0.042	2.78	3.79	3.03	2.78	2.74	926	47
	10	0.417	4.2	5.43	5.78	5.88	5.71	1252	113
	100	4.167	9.67	10.13	10.1	9.86	8.58	781	126
	1000	41.667	17.25	17	18.9	15.8	17.24	741.4	213
Route I	0	0	0	0	0.79	0.88	0.6	452.2	3
	0.5	0.021	0	0.69	0	0	1.29	356	2
	1	0.042	0	0.66	1.45	0	0	588.7	4
	10	0.417	2.77	3.03	2.96	2.7	3.57	427	22
	100	4.167	7.22	5.92	6.33	6	7.4	693.5	76
	1000	41.667	10.52	12	13.8	9.77	11.11	540.5	103

Table 2. Limit of detection (LOD) and dynamic linear range (DLR) comparison of CAN assay to existing immunoassays.

Item	Assay method	LOD (pg/mL)	LOD (fM)	DLR (pg/mL)	Clinically Validated	Ref.
1	Carbon dot-based microplate and microfluidic chip immunoassay	20	833	30-1000	Y	9
2	Gold nanoparticle-based bio barcode amplification assay	0.1	4.2	0.1-500	N	10
3	Fluorescent silver nanoparticle-based immunoassay	8.2	342	10-1000	Y	11
4	Inductively coupled plasma mass spectrometry-based gold nanoparticle immunoassay	7.5	312.5	7.5-75	N	12
5	Gold nanocluster immunoassay	5	208.3	5-1000	Y	13
6	Europium nanoparticle-based microtiter-plate	5	208.3	5-500	N	14
7	Copper nanocluster based immunosensor	23.8	991.7	27-1000	Y	4
8	Europium nanoparticle sandwich immunoassay	0.19	7.91	1-20	N	15
9	Conventional HIV-1 p24 ELISA	10-15	416-625	10-1000	Y	10
10	Fourth-generation HIV screening immunoassays	4-10	166-416	5-1000	Y	16, 17
11	Mercapto succinic acid hydrazide copper (II) (CuL) monolayer modified gold electrode (Au|CuL) based immunosensor	200	8333	500-500000	N	18
12	Acetone-extracted propolis based amperometry immunosensor	6.4	266.7	10-60000	N	19
13	Immunosensor based on directly electroplating an electrode surface with gold nanoparticles using chronoamperometry,	8	333.3	10-100000	N	20
14	P-RGO@Au@Ru-SiO_2 composite based electrochemiluminescence immunosensor	1	41.7	1-10000	N	21
15	Selective detection of Ag^+/ Ag NPs and Cu^{2+}/ Cu^+ using QDs	0.25	10.4	1-100	Y	22
16	Surface-modified mesoporous nanofiber	0.86	35.8	0.86-800	N	23
17	Liposome-amplified photoelectrochemical immunoassay	0.63	26.25	1-50000	N	24
23	Blue emission fluorescent CuNCs linked immunosorbent assay	50	2083	50-1000	N	Present

24	Red emission fluorescent CuNCs linked immunosorbent assay	20	833	20-1000	N	Present
25	ELISA based fourth generation HIV-1 test	10	416.6	10-1000	Y	Present
26	Click chemistry amplified nanopore (CAN) assay	0.5	20.8	0.5-1000	Y	Present

Table 3. Capture rate of multi-level signals obtained from parallel experiments for patients

Patient	VL (copies/mL)	Stage	Capture Rate (min^{-1})			Total Recording time (s)	Nanopore-P24 (fM)
			Pore 1	Pore 2	Pore 3		
1	.	Negative	0	0	1.72	285	1.92
2	.	Negative	0	0.46	0.41	487	0.02
3	.	Negative	0	1.07	0	309	0.31
4	.	Negative	0.93	0	1.4	224.9	1.01
5	.	Negative	0	1.19	1.29	326.3	1.06
6	.	Negative	0.625	0	0	306.4	0.04
7	.	Negative	1.14	0.66	0	346.8	0.43
8	.	Negative	1.17	0	0	279.1	0.42
9	.	Negative	1.31	0	0	209.5	0.65
10	.	Negative	0	2.1	1.96	209.9	7.05
11	.	Negative	2.25	2.4	2	205	15.18
12	0	HIV	3.03	3.46	3.24	296.2	122.74
13	0	HIV	1.66	2.6	0	321.64	10.69
14	0	HIV	1.88	2.52	3.63	291.8	43.35
15	0	HIV	0	0	0	360.6	0.00
16	0	HIV	3.03	2.7	2.77	269.7	38.38
17	0	HIV	0	0.64	0	286.4	0.04
18	0	HIV	3.15	3.22	0	145	39.59
19	0	HIV	0.84	1.01	1.66	400.4	1.97
20	0	HIV	2.3	2.14	2.19	295.5	14.64
21	0	HIV	0	0	0.43	384.7	0.01
22	0	HIV	0.53	1.62	1.42	319.3	2.38
23	0	HIV	1.07	1.169	1.2	394.4	1.18
24	0	HIV	2.71	2.76	2.63	348.6	31.52
25	0	HIV	1.58	2.89	2.43	274.4	21.95
26	0	HIV	0	1.07	0	267.94	0.30
27	0	HIV	1.31	1.45	1.26	306.4	2.17
28	0	HIV	1.94	2.07	1.96	228.5	9.78
29	0	HIV	1.33	2.29	0	305.4	6.26
30	0	HIV	1.28	1.52	1.94	324	4.69
31	0	HIV	0	0.46	0	407	0.01
32	0	HIV	2.094	0.76	1.7	222.5	5.80
33	0	HIV	2	0	2	237.4	6.62
34	0	HIV	1.19	1.02	2.23	256.7	5.73
35	0	HIV	1.72	2.13	0	208	6.07
36	0	AIDS	0	0.93	0	348	0.17
37	0	AIDS	2.5	2.3	2.27	288.7	18.85
38	0	AIDS	1.08	3.33	0.98	304.1	24.00
39	0	AIDS	0	0	0	214.5	0.00
40	0	AIDS	2.02	2.17	1.56	240.7	9.24

41	0	AIDS	0	0	0.71	127.9	0.06
42	0	AIDS	0.88	2	0	161.4	3.45
43	0	AIDS	3.125	3.62	3.05	183.9	67.49
44	0	AIDS	3.84	3.7	4.12	210	128.96
45	LOD~30	HIV	4.47	3.125	3.79	264	137.45
46	LOD~30	HIV	3.33	3.76	3.8	305.5	105.57
47	LOD~30	HIV	3.82	4.65	4.34	230	201.04
48	LOD~30	HIV	4	3.33	3.27	471	98.09
49	LOD~30	HIV	2.5	3.27	2.66	324.4	41.61
50	LOD~30	HIV	3.27	3.82	3.61	429	98.96
51	LOD~30	HIV	1.36	1.36	1.87	518	4.21
52	LOD~30	HIV	4.14	4.34	3.79	599	167.20
53	LOD~30	HIV	4.16	4.59	3.42	591	171.57
54	LOD~30	HIV	3.44	3.42	3.22	453	77.23
55	LOD~30	HIV	3.53	3.72	3.5	340	98.99
56	LOD~30	HIV	3.22	4.22	2.5	442	91.75
57	LOD~30	HIV	1.31	2.31	1.07	410	6.99
58	LOD~30	HIV	3.06	2.91	1.43	453	33.48
59	LOD~30	HIV	3.5	3.23	2.41	631	59.08
60	LOD~30	HIV	1.25	1.77	1.78	196.5	4.90
61	LOD~30	HIV	3.03	3.47	3.125	406.2	62.22
62	LOD~30	HIV	1.62	2.75	2.07	408.4	16.50
63	LOD~30	HIV	2.31	1.81	2.19	214.7	12.70
64	LOD~30	HIV	4.96	5.3	4.41	335	317.60
65	LOD~30	HIV	4.72	4.42	4	304.2	206.85
66	LOD~30	HIV	3.125	3.87	3.63	305.3	92.90
67	LOD~30	HIV	4.28	4.05	4.47	275	184.14
68	LOD~30	HIV	2.6	3.24	2.94	344	47.05
69	LOD~30	HIV	1.72	1.66	2.2	303	8.58
70	LOD~30	HIV	3.28	3.27	2.75	289	58.21
71	LOD~30	HIV	1.8	1.78	1.72	347	6.41
72	LOD~30	HIV	2.58	1.21	3.73	416	48.09
73	LOD~30	HIV	1.21	3.79	4.16	420	99.83
74	LOD~30	HIV	3.63	4	3.5	256.5	108.60
75	LOD~30	HIV	3.92	4.44	3.38	354	139.69
76	LOD~30	HIV	2.56	4.05	1.52	335.4	59.50
77	LOD~30	HIV	2.73	3.47	2.83	223	51.00
78	LOD~30	HIV	3.94	3.06	2.89	187.9	75.38
79	LOD~30	HIV	2.09	2	2.31	259.4	12.99
80	LOD~30	HIV	5.06	4.66	4.61	276	284.47
81	LOD~30	HIV	4.47	4.13	3.45	219.05	153.34
82	LOD~30	HIV	2.3	2.91	2.89	225	33.26
83	LOD~30	HIV	3.92	3.38	4.89	218.3	171.54
84	LOD~30	HIV	3.66	3.44	4.8	263.9	156.01

85	LOD~30	HIV	1.9	0.83	0	266	2.83
86	LOD~30	HIV	4	3.62	3.11	201.2	97.88
87	LOD~30	AIDS	2.31	1.2	1.87	1052	8.78
88	LOD~30	AIDS	4.24	4.28	3.82	458	170.40
89	LOD~30	AIDS	3.68	2.89	3.48	347.4	80.07
90	LOD~30	AIDS	3.96	4.41	3.79	521	162.52
91	LOD~30	AIDS	3.11	3.38	2.46	406	52.89
92	LOD~30	AIDS	4	4.61	3.87	155.5	181.60
93	LOD~30	AIDS	1.71	5	4.34	249.9	178.69
94	LOD~30	AIDS	4.62	4.74	4.54	247.6	251.00
95	LOD~30	AIDS	2.39	2.67	2.41	539	24.48
96	LOD~30	AIDS	3.79	3.33	2.41	361	72.65
97	LOD~30	AIDS	3.3	2.5	2	294.7	33.78
98	LOD~30	AIDS	3.88	4.41	4.88	332.6	213.26
99	LOD~30	AIDS	4.57	4.28	4.54	303.16	218.03
100	67	HIV	5	5.58	5.47	534	470.11
101	119	HIV	3.88	3.64	4	220.3	122.76
102	140	HIV	5.34	5.31	5.88	231.1	493.80
103	345	HIV	5.66	5.67	5.73	307.3	550.62
104	1077	HIV	4.9	5.51	4.81	300.7	362.55
105	1980	HIV	7.1	5.22	8.1	232.2	1277.18
106	4675	HIV	4.42	6.66	6.97	466.9	807.56
107	14165	HIV	4.22	3.03	3.4	287.4	100.08
108	17893	HIV	6.36	4.96	5.88	196	599.29
109	300211	HIV	7.14	10.2	5.26	294.5	2495.91
110	108	AIDS	4.58	4.41	4.42	186.2	218.65
111	230	AIDS	4.93	4.71	5.56	383.5	387.16
112	1023	AIDS	4.81	3.27	4.38	370	197.10
113	8395	AIDS	4.8	4.34	4.95	132	268.31
114	9712	AIDS	5.41	5.12	5.26	464	437.30
115	70223	AIDS	8.1	5.84	6.91	292.9	1305.76
116	167660	AIDS	6.06	5.18	6.97	350.5	818.07
117	222465	AIDS	8.38	7.31	7.14	361.5	1735.86
118	246776	AIDS	5.7	6.32	6.53	461	827.48

References

1. Yan, W. et al. Facile Synthesis of Ultrastable Fluorescent Copper Nanoclusters and Their Cellular Imaging Application. *Nanomaterials* **10** (2020).
2. Jiao, M.X. et al. Ligand-modulated aqueous synthesis of color-tunable copper nanoclusters for the photoluminescent assay of Hg(II). *Microchimica Acta* **187** (2020).
3. Lai, W.F., Wong, W.T. & Rogach, A.L. Development of Copper Nanoclusters for In Vitro and In Vivo Theranostic Applications. *Adv Mater* **32** (2020).
4. Kurdekar, A.D. et al. Computational design and clinical demonstration of a copper nanocluster based universal immunosensor for sensitive diagnostics. *Nanoscale Advances* **2**, 304-314 (2020).
5. Zhou, Q. et al. Supramolecular vesicle: triggered by formation of pseudorotaxane between cucurbit [6] uril and surfactant. *Chem Commun* **47**, 11315-11317 (2011).
6. Moon, K. & Kaifer, A.E. Modes of Binding Interaction between Viologen Guests and the Cucurbit[7]uril Host. *Org Lett* **6**, 185-188 (2004).
7. Wei, X. et al. Molecular mechanisms for delicately tuning the morphology and properties of Fe3O4 nanoparticle clusters. *CrystEngComm* **20**, 2421-2429 (2018).
8. Ye, J., Dong, X., Jiang, H. & Wang, X. An intracellular temperature nanoprobe based on biosynthesized fluorescent copper nanoclusters. *J. Mater. Chem. B* **5**, 691-696 (2017).
9. Chunduri, L.A.A. et al. Development of carbon dot based microplate and microfluidic chip immunoassay for rapid and sensitive detection of HIV-1 p24 antigen. *Microfluidics and Nanofluidics* **20** (2016).
10. Tang, S.X. & Hewlett, I. Nanoparticle-Based Immunoassays for Sensitive and Early Detection of HIV-1 Capsid (p24) Antigen. *Journal of Infectious Diseases* **201**, S59-S64 (2010).
11. Kurdekar, A.D. et al. Fluorescent silver nanoparticle based highly sensitive immunoassay for early detection of HIV infection. *Rsc Advances* **7**, 19863-19877 (2017).
12. He, Q. et al. Detection of HIV-1 p24 antigen using streptavidin-biotin and gold nanoparticles based immunoassay by inductively coupled plasma mass spectrometry. *Journal of Analytical Atomic Spectrometry* **29**, 1477-1482 (2014).
13. Kurdekar, A.D. et al. Streptavidin-conjugated gold nanoclusters as ultrasensitive fluorescent sensors for early diagnosis of HIV infection. *Science Advances* **4** (2018).
14. Liu, J. et al. Development of a microchip Europium nanoparticle immunoassay for sensitive point-of-care HIV detection. *Biosensors & Bioelectronics* **61**, 177-183 (2014).
15. Kurdekar, A., Chunduri, L.A.A., Haleyurgirisetty, M.K., Hewlett, I.K. & Kamisetti, V. Sub-picogram level sensitivity in HIV diagnostics achieved with the europium nanoparticle immunoassay through metal enhanced fluorescence. *Nanoscale Advances* **1**, 273-280 (2019).
16. Speers, D., Phillips, P. & Dyer, J. Combination Assay Detecting both Human Immunodeficiency Virus (HIV) p24 Antigen and Anti-HIV Antibodies Opens a Second Diagnostic Window. *Journal of Clinical Microbiology* **43**, 5397 (2005).
17. Weber, B. Screening of HIV infection: role of molecular and immunological assays. *Expert Review of Molecular Diagnostics* **6**, 399-411 (2006).
18. Gan, N., Li, T.-H., Lei, J.-P., Wang, L.-Y. & Yang, X. Electrochemical Immunosensor for Human Immunodeficiency Virus p24 Antigen Based on Mercapto Succinic Acid Hydrazide Copper Monolayer Modified Gold Electrode. *Chinese Journal of Analytical Chemistry* **36**, 1167-1171 (2008).

19. Kheiri, F., Sabzi, R.E., Jannatdoust, E., Shojaeefar, E. & Sedghi, H. A novel amperometric immunosensor based on acetone-extracted propolis for the detection of the HIV-1 p24 antigen. *Biosensors & Bioelectronics* **26**, 4457-4463 (2011).
20. Zheng, L. et al. A Sandwich HIV p24 Amperometric Immunosensor Based on a Direct Gold Electroplating-Modified Electrode. *Molecules* **17**, 5988-6000 (2012).
21. Zhou, L., Huang, J., Yu, B., Liu, Y. & You, T. A Novel Electrochemiluminescence Immunosensor for the Analysis of HIV-1 p24 Antigen Based on P-RGO@Au@Ru-SiO2 Composite. *ACS Applied Materials & Interfaces* **7**, 24438-24445 (2015).
22. Tang, Z. et al. Fluorescence and visual immunoassay of HIV-1 p24 antigen in clinical samples via multiple selective recognitions of CdTe QDs. *Mikrochim Acta* **188**, 422 (2021).
23. Li, Z. et al. Surface-modified mesoporous nanofibers for microfluidic immunosensor with an ultra-sensitivity and high signal-to-noise ratio. *Biosens. Bioelectron.* **166**, 112444 (2020).
24. Zhuang, J. et al. Liposome-amplified photoelectrochemical immunoassay for highly sensitive monitoring of disease biomarkers based on a split-type strategy. *Biosens Bioelectron* **99**, 230-236 (2018).
