# Supplementary material for: A click chemistry amplified nanopore assay for ultrasensitive quantification of HIV-1 p24 antigen in clinical samples
Source: Nat Commun. 2022 Nov 11;13:6852. doi: 10.1038/s41467-022-34273-x (PMC9651128; doi:10.1038/s41467-022-34273-x)
Supplement: Supplementary file 2 — Reporting Summary [file 41467_2022_34273_MOESM2_ESM.pdf]

# nature portfolio

Corresponding author(s): Chang Liu

Last updated by author(s): Aug 31, 2022

# Reporting Summary

Nature Portfolio wishes to improve the reproducibility of the work that we publish. This form provides structure for consistency and transparency in reporting. For further information on Nature Portfolio policies, see our Editorial Policies and the Editorial Policy Checklist.

## Statistics

For all statistical analyses, confirm that the following items are present in the figure legend, table legend, main text, or Methods section.

| n/a | Confirmed | |
|---|---|---|
| ☐ | ☒ | The exact sample size (*n*) for each experimental group/condition, given as a discrete number and unit of measurement |
| ☐ | ☒ | A statement on whether measurements were taken from distinct samples or whether the same sample was measured repeatedly |
| ☐ | ☒ | The statistical test(s) used AND whether they are one- or two-sided<br>*Only common tests should be described solely by name; describe more complex techniques in the Methods section.* |
| ☒ | ☐ | A description of all covariates tested |
| ☒ | ☐ | A description of any assumptions or corrections, such as tests of normality and adjustment for multiple comparisons |
| ☐ | ☒ | A full description of the statistical parameters including central tendency (e.g. means) or other basic estimates (e.g. regression coefficient) AND variation (e.g. standard deviation) or associated estimates of uncertainty (e.g. confidence intervals) |
| ☐ | ☒ | For null hypothesis testing, the test statistic (e.g. *F*, *t*, *r*) with confidence intervals, effect sizes, degrees of freedom and *P* value noted<br>*Give P values as exact values whenever suitable.* |
| ☒ | ☐ | For Bayesian analysis, information on the choice of priors and Markov chain Monte Carlo settings |
| ☒ | ☐ | For hierarchical and complex designs, identification of the appropriate level for tests and full reporting of outcomes |
| ☒ | ☐ | Estimates of effect sizes (e.g. Cohen's *d*, Pearson's *r*), indicating how they were calculated |

*Our web collection on statistics for biologists contains articles on many of the points above.*

## Software and code

Policy information about availability of computer code

| | |
|---|---|
| Data collection | Clampex 11.0.3 |
| Data analysis | Clampfit 10.7&11, OriginPro 9.0, Graphpad Prism 9.0, MATLAB R2019 a, Python 3.7 modules used for scatter plots and contour plots were Matplotlib and Seaborn's bivariate kernel density estimator |

For manuscripts utilizing custom algorithms or software that are central to the research but not yet described in published literature, software must be made available to editors and reviewers. We strongly encourage code deposition in a community repository (e.g. GitHub). See the Nature Portfolio guidelines for submitting code & software for further information.

## Data

Policy information about availability of data

All manuscripts must include a data availability statement. This statement should provide the following information, where applicable:

- Accession codes, unique identifiers, or web links for publicly available datasets
- A description of any restrictions on data availability
- For clinical datasets or third party data, please ensure that the statement adheres to our policy

The main data supporting the findings of this study are available within the article, the Supplementary Information file, and the Source Data file. The raw datasets generated during the study are not publicly shared but are available for research purposes from the corresponding author upon reasonable request.

# Field-specific reporting

Please select the one below that is the best fit for your research. If you are not sure, read the appropriate sections before making your selection.

- [x] Life sciences
- [ ] Behavioural & social sciences
- [ ] Ecological, evolutionary & environmental sciences

For a reference copy of the document with all sections, see nature.com/documents/nr-reporting-summary-flat.pdf

# Life sciences study design

All studies must disclose on these points even when the disclosure is negative.

| | |
|---|---|
| Sample size | Since this study is for proof-of-concept of a new HIV p24 detection assay, no sample size calculation was performed. A total of 124 human subjects were consecutively recruited for blood samples, and 118 were included in the study. |
| Data exclusions | 6 blood samples were excluded due to contamination, COVID-19, and missing information |
| Replication | All data presented was repeated at least three times. All attempts of replication were successful. |
| Randomization | Samples were allocated into different groups based on viral load values provided by clinical lab as a reference marker. |
| Blinding | Researchers who performed data collection and analysis were blinded to group allocation and detailed patient information. |

# Reporting for specific materials, systems and methods

We require information from authors about some types of materials, experimental systems and methods used in many studies. Here, indicate whether each material, system or method listed is relevant to your study. If you are not sure if a list item applies to your research, read the appropriate section before selecting a response.

## Materials & experimental systems

| n/a | Involved in the study | |
|---|---|---|
| [ ] | [x] | Antibodies |
| [x] | [ ] | Eukaryotic cell lines |
| [x] | [ ] | Palaeontology and archaeology |
| [x] | [ ] | Animals and other organisms |
| [ ] | [x] | Human research participants |
| [ ] | [x] | Clinical data |
| [x] | [ ] | Dual use research of concern |

## Methods

| n/a | Involved in the study | |
|---|---|---|
| [x] | [ ] | ChIP-seq |
| [x] | [ ] | Flow cytometry |
| [x] | [ ] | MRI-based neuroimaging |

## Antibodies

| | |
|---|---|
| Antibodies used | anti-HIV-1 p24 antibody [5] (ab63958); anti-HIV-1 p24 antibody [38/8.7.47] (ab9044); biotin anti-HIV-1 p24 antibody (ab68617); HIV-1 p24 ELISA kit (ab218268). All of these regents were purchased from Abcam. All antibodies were used directly or diluted according to the manufacturer's specifications. |
| Validation | All validation data of antibodies used can be found on ABCAM website by product numbers: https://www.abcam.com/products?selected.classification=Primary +antibodies&selected.brand=RabMAb&gclid=CjwKCAjwz_WGBhA1EiwAUAxIcdtLye9ECgEFdAHI5wqDUHksTUcs0cniRndY4dJnG78KASmvPhaN2BoC6WEQAvD_BwE&gclsrc=aw.ds |

# Human research participants

Policy information about studies involving human research participants

| | |
|---|---|
| Population characteristics | Among the 118 eligible subjects (age 20-78), 86 (73%) are male. |
| Recruitment | Serum samples and associated clinical data were collected using a University of South Carolina IRB approved protocol (IRB ID: Pro00083810) from enrolled adults who visited Prisma Health Richland Hospital from March 2020 to October 2021 for medical evaluation. All study subjects were evaluated by clinicians in the Prisma Health Immunology Center and were enrolled only after written informed consent was obtained. Samples were excluded if there were possible contamination, known COVID-19 infection, or missing information. No further selection has been made to either positive or negative samples. |
| Ethics oversight | This study was approved by the University of South Carolina Institutional Review Board for Human Research (Approval No. Pro00083810). All research was performed in accordance with relevant guidelines and regulations. All participants have provided informed consent. Participants were not compensated. Study results were not used in any ways in clinical procedures and were not made available to participants. |

Note that full information on the approval of the study protocol must also be provided in the manuscript.

# Clinical data

Policy information about clinical studies

All manuscripts should comply with the ICMJEguidelines for publication of clinical research and a completedCONSORT checklist must be included with all submissions.

| | |
|---|---|
| Clinical trial registration | N/A |
| Study protocol | Not clinical trial. Study protocols can be obtained through IRB or by contacting the corresponding author. |
| Data collection | Blood samples and clinical data were collected at Prisma Health Richland Hospital from March 2020 to October 2021. Experimental data was collected at University of South Carolina Biosensing and Biomarker Research Lab from March 2020 to December 2021. |
| Outcomes | The primary outcome is the percentage of patients detected by the CAN assay among those undetectable by benchmark assays. The secondary outcome is the limit of detection of the CAN assay for p24 in human serum. These measures were assessed by side-by-side comparison to benchmark p24 assays (fluorescence assay and ELISA). |
